# Left vs. right radial approach for coronary catheterization: Relation to age and severe aortic stenosis

**DOI:** 10.3389/fcvm.2022.1022415

**Published:** 2022-10-31

**Authors:** Maximilian Will, Thomas W. Weiss, Michael Weber, Chun Shing Kwok, Josip A. Borovac, Gudrun Lamm, Markus Unterdechler, Simone Aufhauser, Jim Nolan, Julia Mascherbauer, Konstantin Schwarz

**Affiliations:** ^1^Department of Internal Medicine 3, University Hospital St. Pölten, Karl Landsteiner University of Health Sciences, Krems, Austria; ^2^Karl Landsteiner Institute for Cardiometabolics, Karl Landsteiner Society, St. Pölten, Austria; ^3^Medical School, Sigmund-Freud University, Vienna, Austria; ^4^Division Biostatistics and Data Science, Department of General Health Studies, Karl Landsteiner University of Health Sciences, Krems, Austria; ^5^Department of Cardiology, Royal Stoke University Hospital, Stoke-on-Trent, United Kingdom; ^6^School of Medicine, Keele University, Keele, United Kingdom; ^7^Clinic for Heart and Vascular Diseases, University Hospital of Split, Split, Croatia; ^8^CDL VaSiCS, LIT CPS Lab, Johannes Kepler University Linz, Linz, Austria

**Keywords:** coronary angiography (CAG), radial access, aortic unfolding, elderly patients, aortic stenosis (AS)

## Abstract

**Background:**

Old age and the presence of aortic stenosis are associated with the unfolding of the intrathoracic aorta. This may result in increased difficulties navigating catheters from the right compared to the left radial approach.

**Objective:**

To investigate whether increasing age or presence of severe aortic stenosis was associated with increased catheterization success rates from left (LRA) compared to right radial artery approach (RRA).

**Methods:**

We compared coronary angiography success rates of RRA and LRA according to different age groups and in a subgroup of patients with severe aortic stenosis.

**Results:**

A total of 21,259 coronary angiographies were evaluated. With increasing age, the first pass success rate from either radial access decreased significantly (*p* < 0.001). In patients aged <85 years, there was no difference between LRA and RRA. However, in patients aged ≥85 years, LRA was associated with significantly higher success rates compared to RRA (90.1 vs. 82.8%, *p* = 0.003). Patients aged ≥85 years received less contrast agent and had shorter fluoroscopy time when LRA was used [86.6 ± 41.1 vs. 99.6 ± 48.7 ml (*p* < 0.001) and 4.5 ± 4.1 min vs. 6.2 ± 5.7 min (*p* < 0.001), mean (±SD)]. In patients with severe aortic stenosis (*n* = 589) better first pass success rates were observed *via* LRA compared to the RRA route (91.9 vs. 85.1%, *p* = 0.037).

**Conclusion:**

LRA, compared to RRA, is associated with a higher first-pass catheter success rate for coronary artery angiography in patients aged ≥85 years and those with severe aortic stenosis.

## Introduction

Coronary angiography and percutaneous coronary intervention (PCI) play a major role in the diagnosis and treatment of coronary artery disease (CAD) (1–3). Since the first transradial procedure in 1989 (4), radial artery replaced femoral approach as the first access site choice for most coronary procedures. The shift toward radial access was possible due to evolution of smaller size equipment and its popularity was driven by its lower bleeding risk compared to femoral access (4).

In current clinical practice, radial access is performed primarily from the right radial artery. The reason is an ergonomic catheterization table setup for right-handed operators. An exception to this practice is coronary angiography in patients with previous coronary artery bypass grafting (CABG) with a left internal mammary artery graft where right-radial approach is associated with low success rates (5). While some authors report shorter fluoroscopy time for LRA when compared to RRA (6, 7), others suggest no significant differences between left and right radial access in terms of success rates, amount of contrast used, or complications (8, 9). Small, randomized trials reported less radiation exposure to the operator with LRA compared to RRA (6, 10).

We hypothesize that specifically in elderly patients an initial LRA, compared to the RRA approach might result in higher success rates.

**Figure 1:**
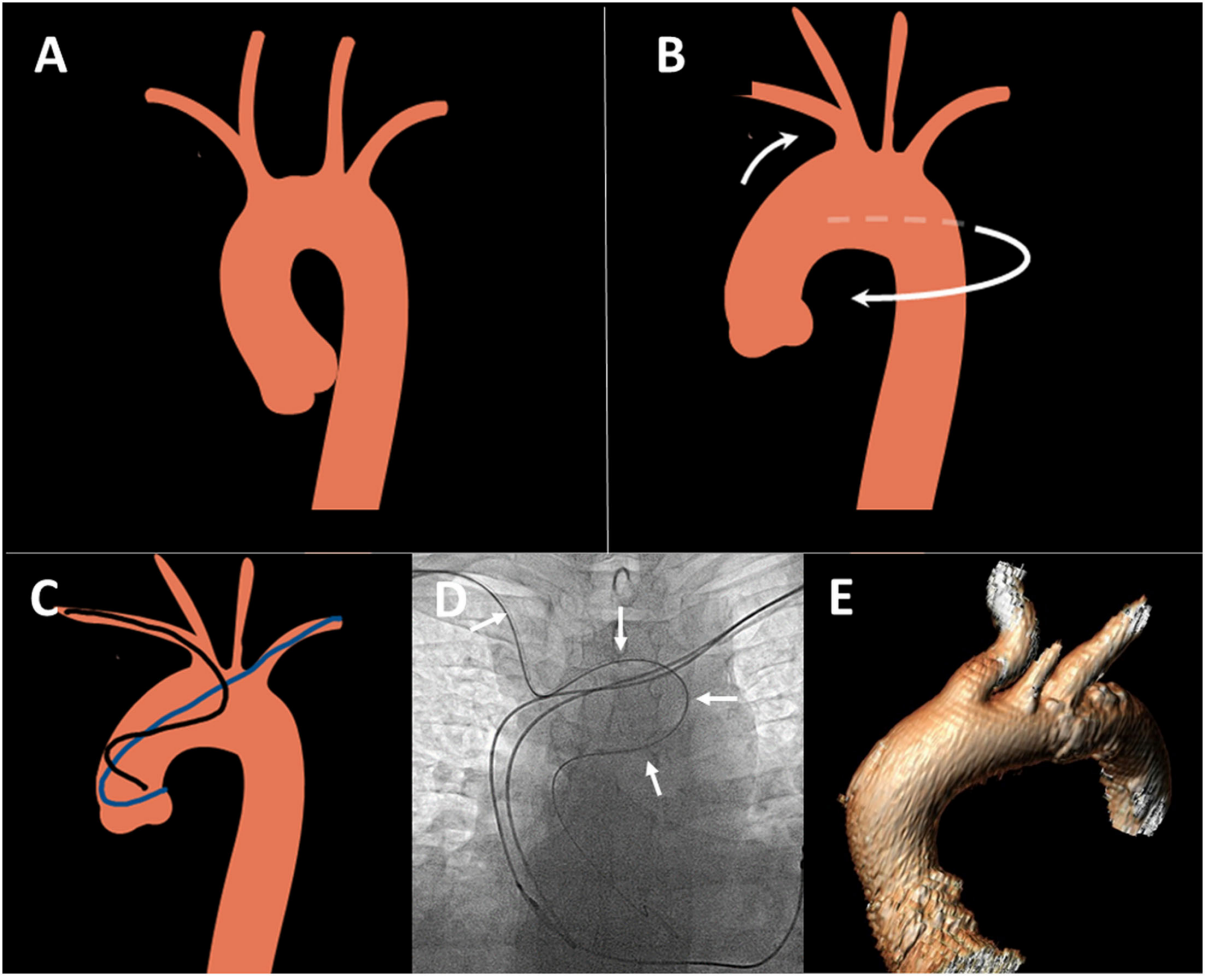
Aortic unfolding and its consequences for transradial angiography. **(A)** Normal aortic arch anatomy: **(B)** Aortic unfolding leading to anti-clockwise rotation, elongation of aortic arch, posterior displacement and tortuosity of right radial and innominate artery (white arrows). **(C)** Unfavorable catheter position from right radial (black) compared to left radial (blue) access route in unfolded thoracic aorta. Figures created using smart.servier.com image bank. **(D)** Example of tortuous catheter route in unfolded aorta in elderly patient from right radial route, white arrows: EBU catheter. **(E)** 3D reconstruction of the unfolded aorta and posteriorly displaced innominate artery in a patient with severe aortic stenosis.

There are a few anatomical differences between LRA and RRA access routes, which are hypothesized to be a consequence of age-related changes in aortic arch geometry (11). Aortic unfolding is a condition described as the elongation and increased curvature of the aortic arch (Figure 1) (8). It is associated with increasing age, proximal aortic stiffness (11), body surface area, hypertension, and increased coronary artery calcification (9). Furthermore, right subclavian tortuosity was more frequently observed compared to the left side (12, 13). These age-related aortic arch changes could lead to a lower success rate when performing diagnostic angiography from RRA and **one** could assume that it may be easier to overcome aortic unfolding due to better anatomical angulation *via* the LRA route (Figure 1).

To determine whether there is any age-related difference in LRA compared to the RRA approach, we evaluated the success rates of both routes in different age groups and a population with severe aortic stenosis. Our primary hypothesis stated that in the elderly population LRA, compared to RRA will be associated with a higher success rate to engage coronary arteries. Our second hypothesis was that a higher success rate will also be achieved *via* the LRA approach in a population with severe aortic stenosis. If the primary hypotheses were proven correct, we planned further analysis, whether a primary LRA use could be associated with less fluoroscopy time and saving of contrast volume.

## Materials and methods

This retrospective observational study was approved by the Karl-Landsteiner Scientific Integrity and Ethics Commission (ethics commission number 1056/2021). The reporting of this study is in accordance with the STrengthening the Reporting of OBservational studies in Epidemiology (STROBE) recommendations (14).

All consecutive patients who underwent cardiac catheterization *via* primary radial access between January 2014 and November 2021 were identified from the local database at the Sankt Pölten University Hospital, Austria. Data extraction was done by MaWi and MU independently on two different occasions yielding the same results and a data quality check was independently performed by KS and MaWi.

To compare LRA vs. RRA success rates, we stratified the whole population into subgroups according to their age and compared if there was a difference between the right and left radial approaches in terms of success. A first-pass success was defined as a successful coronary angiography without the need to switch to an alternative access route. The stratification into age groups was set by decades in the younger patients, and in half decades in patients above the age of 70 years, where we hypothesized the impact of aortic elongation on the success rates *via* RRA.

A second analysis compared LRA and RRA first-pass success rates in a population of patients with severe aortic stenosis. These patients were selected from our database under the coding of “Transcutaneous aortic valve replacement (TAVR) evaluation”. All these patients were previously diagnosed with severe aortic stenosis by echocardiography and underwent elective coronary angiography prior to heart team discussion regarding the best treatment strategy (transcutaneous aortic valve replacement vs. surgical valve replacement). Most of the aortic stenosis patients received an aortogram to assess iliac and femoral arteries for TAVR access, resulting in extra radiation dose and contrast volume.

For the comparison of fluoroscopy time, contrast volume, and number of used catheters, only patients with diagnostic procedures were included. Contrast volume, fluoroscopy time, and the number of used catheters were compared for all angiographies, which were initially started by either radial artery access (regardless of first pass success and the possible need for switch to an alternative route). All diagnostic procedures, which in the same session were followed by PCI, were excluded. Furthermore, diagnostic procedures in patients with CABG were also excluded.

### Statistical analysis

All statistical tests were performed by MiWe using the SPSS software (IBM, Version 27.0, IBM, Armonk, NY). Due to the large sample size, we did not use statistical tests for normal distribution but used histograms and QQ-plots to identify relevant deviations from a normal distribution. In cases of skewed data, we used median as well as first and third quartiles (IQR, interquartile range) as descriptive statistics. Nominal data were described using absolute frequencies and percentages. To compare contrast volumes and fluoroscopy times of the left- vs. right-radial approach, Mann-Whitney U-tests were used due to skewed data. The success rates of the **two** approaches were compared using crosstabs and chi^2^ tests. To identify predictors of success for the left and right radial approach, multiple logistic regressions were used. All tests were two-tailed and *p* < 0.05 was considered statistically significant. Normally distributed data were expressed as mean ±SD and non-normally distributed data as median (IQR).

## Results

A total of 21,259 coronary angiographies with primary radial access were included in our analysis. The mean age of the patients was 67.8 ± 11.7 years and 14,024 (66%) were male. The majority were elective cases (16,493, 77.6% respectively), whereas 4,758 (22.4%) angiographies were undertaken due to acute coronary syndrome (ACS).

Multiple binary logistic regression revealed that the first-pass success rate of transradial access was independently affected by increasing age, gender, and the side of access [*p* < 0.001, *p* = 0.028, *p* = 0.017, respectively (Table 1)]. Increasing age was the strongest predictor of failure to successfully carry out trans-radial angiography by the first pass, whereas female gender and RRA predicted slightly less successful first-pass success.

**Table 1:** Predictors of transradial success in a logistic regression model.

**Independent variable**	**Odds ratio (95% CI)**	***p***
Sex	1.1 (1.0–1.2)	0.028
Side	0.8 (0.8–1.0)	0.017
Age	1.0 (0.9–1.0)	<0.001

**Figure 2:**
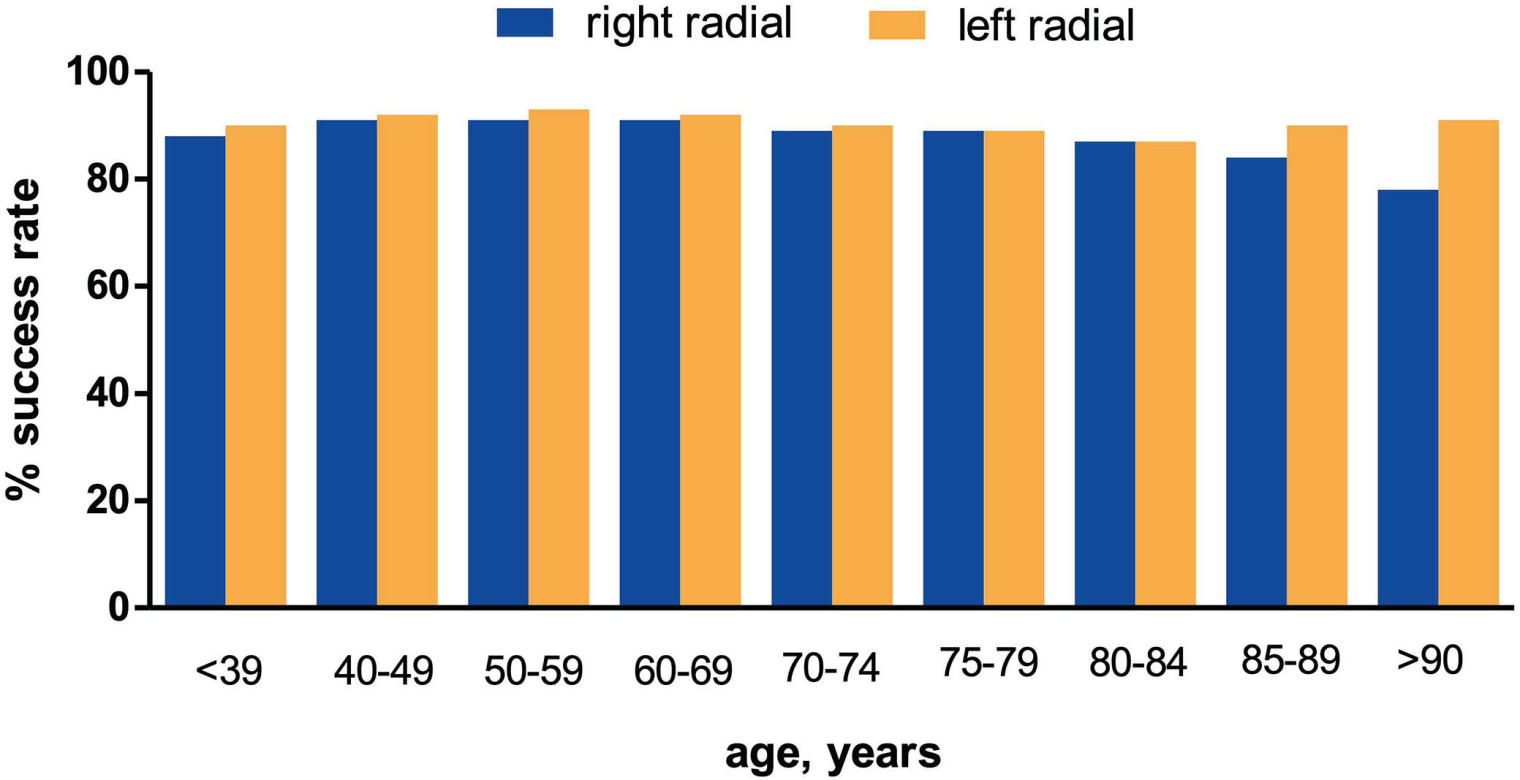
Age-dependent comparison of coronary angiography success rates (RRA vs. LRA route).

Success rates for RRA and LRA stratified by different age groups are shown in Table 2 and Figure 2. The reduction in success rates with RRA was most evident among patients of age 85 or older, whereas success rates *via* LRA remained stable in this age group (Figure 2). For these elderly patients, the rate of first-pass success for LRA compared to RRA was 90.1 vs. 82.8% (*p* = 0.003), respectively. In patients aged <85 years, there was no difference in the first-pass success rate comparing RRA vs. LRA [13,551 (90.0%) vs. 4,614 (90.8 %), *p* = 0.11]. When the study population was divided into more detailed age groups, the age group between 50 and 59 years showed a statistically greater proportion of success with LRA compared to RRA that was statistically significant (*p* = 0.031). We have no explanation for this statistical finding, which is at odds with findings in other similar age groups, but the clinical significance of a 2% side difference is clinically negligible and could be driven by large sample size.

**Table 2:** Comparison of coronary angiography success rates RRA vs. LRA route—divided by age groups and for severe aortic stenosis population.

**Age**	**RRA**	**RRA**	**LRA**	**LRA**	***p***
**(y)**	**success,**	**success,**	**success,**	**success**	
	***n*** **(total)**	**%**	***n*** **(total)**	**%**	
<39	155 (176)	88.1	69 (77)	89.6	0.832
40–49	842(929)	90.6	294 (321)	91.6	0.654
50–59	2,686 (2,961)	90.7	841(904)	93.0	0.031^*^
60–69	3,762 (4,133)	91.0	1,210 (1,317)	91.9	0.371
70–74	2,230 (2,494)	89.4	800 (881)	90.8	0.271
75–79	2,377 (2,655)	89.5	846 (948)	89.2	0.806
80–84	1,498 (1,712)	87.5	554 (636)	87.1	0.834
85–90	568 (677)	83.9	214 (238)	89.9	0.025^*^
>90	112 (144)	77.8	49 (54)	90.7	0.041^*^
Total	14,232 (15,883)	89.6	4,877 (5,376)	90.7	0.020^*^
< 84	13,551 (15,061)	90.0	4,614 (5,084)	90.8	0.109
85+	680 (821)	82.8	263 (292)	90.1	0.003^*^
AS	509 (589)	85.1	125 (136)	91.1	0.037^*^

RRA, Right radial access; LRA, Left radial access;

* p < 0.05, AS severe aortic stenosis population.

Fluoroscopy time and contrast volume increased with age and were affected by the choice of access side (Table 3).

**Table 3:** Contrast volume and fluoroscopy times^*^.

**>85 years of age**	***n***	**RRA**	**LRA**	***p***
Contrast volume ml, median (IQR)	586	94 (62–130)	77 (55–110)	<0.001
Fluoroscopy time min, median (IQR)	586	4.4 (2.4–7.8)	3.2 (1.7–5.9)	<0.001
**Aortic stenosis**		**RRA**	**LRA**	**p**
Contrast volume ml, median (IQR)	671	116.5 (93–14)	116.0 (94–160)	0.861
Fluoroscopy time min, median (IQR)	671	5.3 (3.1–0.4)	6.1 (3.0–10.5)	0.716

* Results after exclusion of PCIs, CABG-angiographies, and statistical outliers (>100 min fluoroscopy time and 500 ml contrast volume); IQR, interquartile range.

Patients who underwent LRA received significantly lower median contrast volume (77 vs. 94 ml, *p* < 0.001). Every 1-year age increase was associated with the additional use of a 0.3 ml contrast agent. This increase was more evident in men (9.9 ml more than in women) and with RRA (3.0 ml more than with LRA). In case of **first** pass failure, an additional 17.2 ml was required. These analyses concerning contrast use were adjusted for age, gender, and first-pass failure. In non-adjusted analysis, LRA compared with RRA was associated with an average reduction in contrast volume of 33 ml (95%CI: 5.346–1.330).

Moreover, LRA was associated with a significantly shorter median fluoroscopy time (3.2 vs. 4.4 min, *p* < 0.001) in our study population. On average, an increase in age by one year was associated with a 0.2-min longer fluoroscopy time for the procedure. This increase was pronounced in men (1.0 min more than in women) and with RRA (1.5 min more than with LRA). In case of first pass failure, RRA required a 2.5-min longer fluoroscopy time when compared to LRA. These findings concerning fluoroscopy time were adjusted for age, gender, and first-pass failure. In non-adjusted analysis, LRA compared with RRA was associated with an average reduction of fluoroscopy time of 1.6 min (95%CI: 2.03–1.1).

The number of used diagnostic material was numerically marginally different between right and left radial access groups, favoring the latter [*n* = 11,352, 2.58 (2.57–2.60) vs. 2.52 (2.48–2.56), respectively, *p* = 0.002, mean (95% CI)]. This finding was numerically more pronounced in patients older than 85 years, and probably due to the smaller sample size did not reach statistical significance [*n* = 545, 2.90 (2.82–2.97) vs. 2.75 (2.57–2.93), respectively, *p* = 0.127, mean (95% CI)].

**Figure 3:**
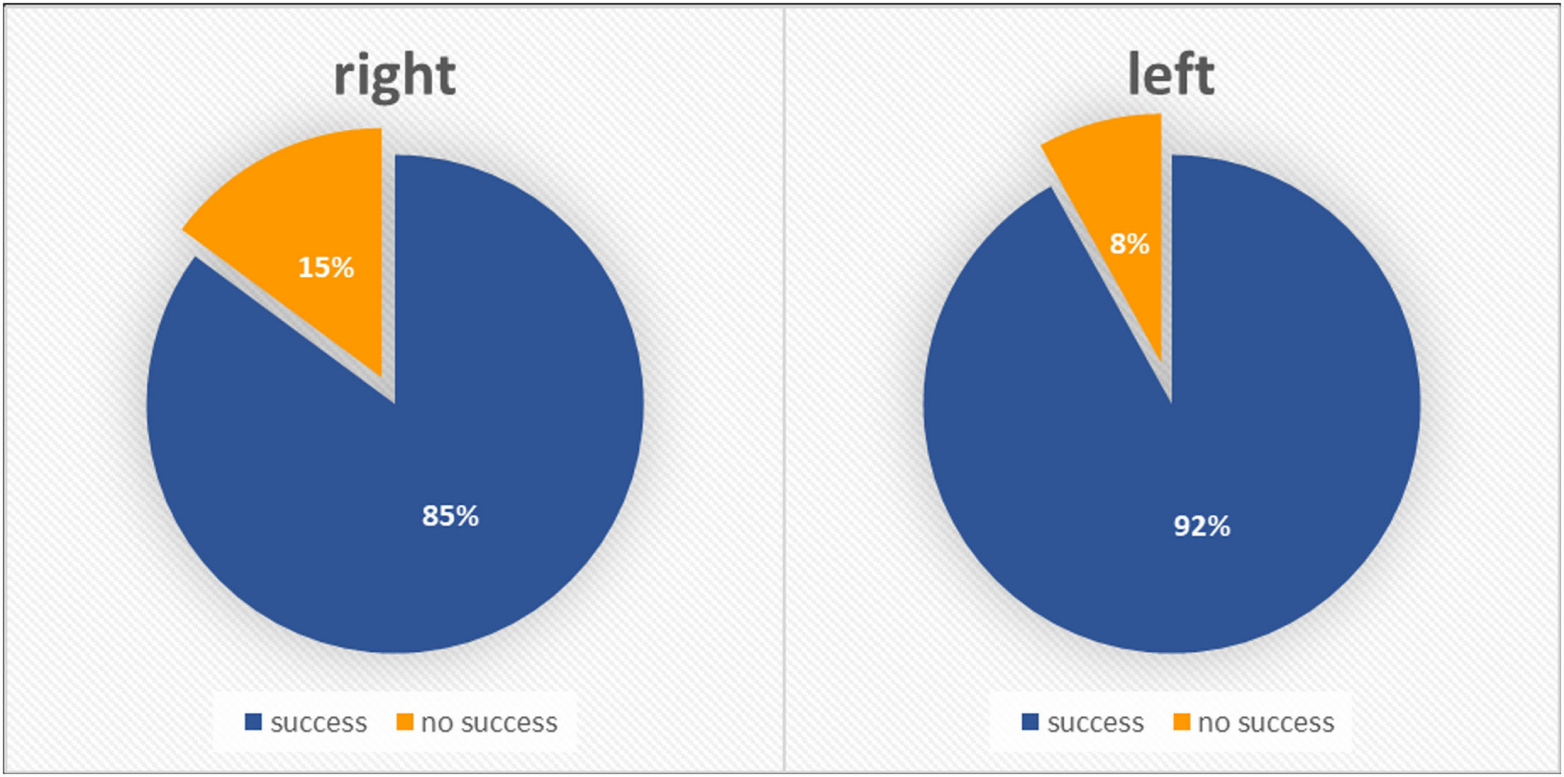
Severe aortic stenosis population–radial approach success rates according to initial side choice.

In the subgroup of patients with severe AS, LRA led to significantly higher success rates compared with RRA (91.9 vs. 85.1%, *p* = 0.037 (Figure 3; Table 2). Fluoroscopy time and used contrast volume did not differ between the two groups in this cohort [8.2 (7.5) min vs. 8.5 (10.3) min, *p* = 0.716 and 128.7 (52.4) vs. 129.8 (79.0) ml, *p* = 0.861, respectively, mean (SD)]. In the subgroup of patients with severe aortic stenosis, the mean age was 81.4 [5.6]. On average, the aortic valve area prior to TAVR was 0.72 cm^2^ and AV mean was 44.9 mmHg.

## Discussion

This study has several key findings. First, left radial access compared to the right radial approach was associated with a higher first pass success rate in the elderly population (≥85 years of age) and in a population with severe aortic stenosis. In the elderly population, the left radial approach resulted in shorter fluoroscopy times and lower contrast volume use. Radial approach success rates from either side decreased with older age and were lower in female compared to male patients.

There is strong evidence supporting radial over femoral access safety advantage in the elderly population (15–17). In 2009, Dehghani et al. postulated three independent risk factors of transradial PCI failure: age above 75 years, prior coronary artery bypass graft surgery, and short stature (18). In our study, increasing age was the strongest predictor of failure to successfully navigate cardiac catheter *via* radial access, hence making the aspiration of achieving safe radial access in every geriatric patient potentially more challenging (19).

Notably, congenital coronary artery disease or malformation may have influenced coronary artery catheterization. Unfortunately, there is no available data concerning this topic in our study population. Nevertheless, according to previous literature, the prevalence of coronary anomalies in most populations is quite low and reported only in 0.2–2% of all coronary angiographies (20).

Our findings of worse radial success in women are supported by the findings of existing literature. Radial access in women has been reported to be affected by smaller vessel calibers and higher rates of radial artery spasm, which can result in procedure failure and high crossover rates to femoral access (18, 21). Pandie et al. (22) reported a 2-fold higher failure of first pass radial success in a pre-specified subgroup analysis of the randomized, multicenter RIVAL (Radial vs. Femoral Access for Coronary Intervention) trial (23). Crossover from radial to femoral approach was reported with 11.1% in women and 6.3% in men, and this is a consequence of higher rates of radial artery spasm (9.5% in women vs. 3.3% in men).

We present a novel finding identifying the age of 85 years (or older) as a cut-off age when there was greater coronary artery catheterization success *via* the LRA compared to RRA. In addition, LRA was associated with a decrease in fluoroscopy time and used contrast agents in this population. Previous randomized trials compared the efficacy of the right- vs. left-radial approach for coronary angiography. Dominici et al. demonstrated a reduction of fluoroscopy time and decreased the number of catheters used *via* LRA in a prospective trial including over 1,000 patients (24). Surprisingly, no significant difference in the amount of used contrast agent was observed between the groups. Another prospective trial by Norgaz et al. (25) randomized 1,000 patients to RRA or LRA and fluoroscopy time was found to be significantly shorter *via* LRA compared with RRA. However, no significant difference comparing LRA and RRA in terms of success rate, procedural time, amount of contrast used, or the number of diagnostic catheters used In the TALENT-trial (13), left radial access for diagnostic angiography was associated with significantly lower fluoroscopy time and this was more evident among lower experienced operators and elderly patients (>70 years) according to prespecified subgroup analyses. However, the benefits of LRA were not consistent across the literature, and differences in procedural and fluoroscopy times could not be demonstrated in small randomized series of 100 octogenarian patients (26). A large meta-analysis, including 14 studies by Xia et al. (26) on a total of 6,870 patients, reported a significant reduction in fluoroscopy times, less contrast volume, and fewer catheter numbers used *via* the left radial route, but no significant difference in procedural failure or procedural times when comparing the two approaches.

Interestingly, whereas most previous studies concentrated on fluoroscopy times, numbers of catheters used, or contrast volume used, they either rarely reported or failed to demonstrate a difference in first pass success rates comparing the two approaches.

The success rates of the two different radial approaches depending on different age distributions were rarely reported. To the best of our knowledge, our study with over 21,000 coronary angiographies presents by far the largest original data set to date, which addresses the question of the LRA vs. RRA approach for successful coronary angiography in different age groups. We identified that the LRA was superior to the RRA for the patients in the age group of >85 years. The most likely explanation is difficult catheter navigation due to tortuosity in the right subclavian artery, which was previously more frequently reported when compared to the left subclavian artery (13, 26). Thoracic aortic unfolding was described with increasing age (11), and is most likely preventing a smooth path of the catheter on its way from the right radial artery to the aortic root in elderly patients.

Not only age but also calcific aortic disease seems to play a major role in the pathophysiology of aortic unfolding. Aortic stenosis is associated with post-stenotic ascending aorta dilation and geometric changes in intrathoracic aorta (27). This may negatively affect the right radial catheter route. To the best of our knowledge, we are the first group to demonstrate that the right radial success rate decreased, whereas the left radial success rates remained stable in patients with severe aortic stenosis. There was no difference in fluoroscopy time or contrast volume used in the severe aortic stenosis population. We have no explanation for this; however, presume that fluoroscopy times and especially contrast volume comparison in the two groups is somewhat difficult due to varying practice and volumes injected into the abdominal aorta for femoral and iliac arteries assessment prior to femoral TAVR access route planning. The contrast volume injected into descending aorta is frequently altered according to patient size and kidney function, and was recently frequently omitted with good CT angiography planning. In the future, large prospective randomized trials and other real-world registry data are warranted to confirm our results.

### Study limitations

This study has several limitations. First, it is a single-center study and the practice in Austria may not be the same in other countries. Second, the study took place over 7 years and some of the procedures were undertaken at a time when operators were transitioning from a routine femoral to radial approach. Third, there is no data on the experience of the operator performing the procedures as both experienced and training operators carried out the procedures at our institution. However, the experience of operators and their preferred access side choice should not affect the fact that older age would be associated with different outcomes if the operators stuck to their preferred initial side choice across the entire age range.

Another limitation is that our aortic stenosis population data apply to elderly patients with degenerative aortic stenosis and cannot be extrapolated to other etiologies (e.g., bicuspid aortic valve).

Finally, we initially planned to collect data and adjust to the presence of hypertension or body size which may impact the thoracic aorta and subclavian artery anatomy, but these data were incomplete so could not be reliably included.

Despite the limitations mentioned above, our data set offers real-world insight into the practice, avoiding other biases associated with prospective randomized trials, regarding the selection of a certain patient population or certain operator experience. The unselected nature of our documented procedures makes the information obtained generally transferable to most settings.

## Conclusion

Compared to the RRA, the use of the LRA approach was associated with the higher success of coronary artery angiography in patients above 85 years of age and those with severe AS. These selected patients may benefit from an initial LRA approach to reduce the need to change the access side.

## Data availability statement

The raw data supporting the conclusions of this article will be made available by the authors, without undue reservation.

## Ethics statement

The studies involving human participants were reviewed and approved by Kommission für Scientific Integrity und Ethik der Karl Landsteiner Privatuniversität, Krems an der Donau. Written informed consent for participation was not required for this study in accordance with the national legislation and the institutional requirements.

## Author contributions

MWi and KS designed the study, validated and interpreted the results, wrote and drafted the manuscript, and wrote the final manuscript. MU, KS, and MWi created the database. MWe analyzed the data. GL collected the AS population demographic data. TW, CK, JB, SA, JN, GL, and JM revised and edited the manuscript. All authors have read and agreed to the published version of the manuscript.

## Funding

We acknowledge the support of the Open Access Publishing Fund of Karl Landsteiner University of Health Sciences, Krems, Austria.

## Conflict of interest

The authors declare that the research was conducted in the absence of any commercial or financial relationships that could be construed as a potential conflict of interest.

## Publisher's note

All claims expressed in this article are solely those of the authors and do not necessarily represent those of their affiliated organizations, or those of the publisher, the editors and the reviewers. Any product that may be evaluated in this article, or claim that may be made by its manufacturer, is not guaranteed or endorsed by the publisher.
