# Supplementary material for: Left vs. right radial approach for coronary catheterization: Relation to age and severe aortic stenosis
Source: Front Cardiovasc Med. 2022 Oct 31;9:1022415. doi: 10.3389/fcvm.2022.1022415 (PMC9662167; doi:10.3389/fcvm.2022.1022415)
Supplement: Supplementary file 1 [file Table_1.docx]

# Supplementary material

**Supplementary Table 1-**Aortic stenosis population demographics

| Severe AS population | Total |
| --- | --- |
| Total | 462 |
| Female % (n) | 53.5% |
| Age (years [SD]) | 81.4 [5.6] |
| Height (cm [SD] | 165.9 [10.7] |
| Weight (kg [SD] | 77.1 [18.1] |
| BMI [SD] | 28.0 [9.2] |
| Smoking | 6.5% (30) |
| Hyperlipidemia | 72.0% (333) |
| Creatinine clearence [SD] | 54.8 [20.0] |
| Diabetes | 34.8% (161) |
| AV-mean (mmHg) [SD] | 44.9 [16.4] |
| AVA (cm^2^) [SD] | 0.72 [0.3] |
